# When Two Worlds Collide: A Rare Case of Multiple Myeloma With Extramedullary Plasmacytoma

**DOI:** 10.7759/cureus.50058

**Published:** 2023-12-06

**Authors:** Jorge Nadal Bosch, Mario Moya, Samuel Serna, Lee Drinkard, Javier Malcolm

**Affiliations:** 1 Internal Medicine, University of Texas Rio Grande Valley, Edinburg, USA; 2 Radiology, Doctors Hospital at Renaissance, Edinburg, USA; 3 Oncology, Doctors Hospital at Renaissance, Edinburg, USA; 4 Medical Information, Doctors Hospital at Renaissance, Edinburg, USA

**Keywords:** plasmacytoma treatment, solitary bone plasmacytoma, extramedullary multiple myeloma, extra-medullary plasmacytoma, diagnosis of multiple myeloma

## Abstract

In this case report, we discuss the presentation, diagnosis, and management of a 67-year-old gentleman with stage II multiple myeloma with concurrent biopsy-proven bone plasmacytoma and why it is important to understand the molecular intricacies of these disorders. We emphasize the critical role of radiology in identifying, characterizing, and managing these lesions. Furthermore, we shed light on the critical differentiation between solitary extramedullary plasmacytoma and multiple myeloma and discuss treatment modalities for both conditions.

## Introduction

Plasma cell neoplasms, also known as plasma cell dyscrasias, encompass a group of disorders characterized by the proliferation of a single clone of plasma cells, typically producing monoclonal immunoglobulin. These conditions can manifest as solitary lesions, such as solitary plasmacytoma, or multiple lesions, as seen in multiple myeloma [1]. The precise factors contributing to the development of multiple myeloma versus plasmacytoma remain incompletely understood, with some evidence suggesting differences in cellular adhesion molecules or chemokine receptor expression profiles among malignant plasma cells. Multiple myeloma is a cancer characterized by the accumulation of abnormal plasma cells in the bone marrow, leading to bone lesions, anemia, kidney damage, and elevated levels of monoclonal proteins. It is a systemic condition with widespread impact. In contrast, plasmacytoma is a localized form, presenting as a single mass or tumor, either in a bone (solitary osseous plasmacytoma) or soft tissues (extramedullary plasmacytoma) [2]. Plasmacytoma may precede the development of multiple myeloma, but its effects are generally more contained, with less systemic impact [3]. The distinction between these conditions is essential for determining appropriate management and monitoring strategies. Regular medical assessments are crucial to identify any progression from plasmacytoma to multiple myeloma.

## Case presentation

A 67-year-old gentleman presented to the emergency department with a 1½-month history of bilateral lower extremity weakness, numbness, and mid-lower back pain. His symptoms had progressed to the point of impaired ambulation, and he experienced a fall due to leg weakness. On physical examination, the patient displayed alertness, intact cranial nerve function, and symmetric motor strength (5/5) in the upper extremities but significant weakness (1/5) in the lower extremities. Sensation remained intact, with noted paraspinal tenderness upon palpation throughout the spine. Laboratory studies are shown below (Table 1).

**Table 1:** Laboratory results Laboratory studies revealed leukopenia, normocytic anemia, thrombocytopenia, hypercalcemia, elevated total protein, and hypoalbuminemia. Of note, an elevated protein gap was noted, along with evidence of monoclonal gammopathy. WBC: white blood cell count, MCV: mean corpuscular volume, CO2: carbon dioxide, BUN: blood urea nitrogen, AST: aspartate transaminase, ALT: alanine transaminase, PT: prothrombin time, INR: international normalized ratio, PTT: partial thromboplastin time, PTH: parathyroid hormone, PTH-RP: parathyroid-related hormone, IgA: immunoglobulin A, IgM: immunoglobulin M, IgG: immunoglobulin G, IgE: immunoglobulin E

WBC	4.70 th/uL	4.0-11.0 th/uL	
Hemoglobin	11.1 g/dL	12.0-15.5 g/dL	
Hematocrit	31.70%	36.0-46.0%	
MCV	89.8 fL	80.0-100.0 fL	
Sodium	128 mmol/L	135-145 mmol/L	
Potassium	4.0 mmol/L	3.5-5.0 mmol/L	
Chloride	98 mmol/L	98-107 mmol/L	
CO2	26 mmol/L	23-29 mmol/L	
Creatinine	0.9 mg/dL	0.6-1.3 mg/dL	
BUN	16 mg/dL	7-20 mg/dL	
Calcium	12.9 mg/dL	8.5-10.5 mg/dL	
Glucose	155 mg/dL	70-99 mg/dL	
AST	30 IU/L	10-40 IU/L	
ALT	19 IU/L	7-56 IU/L	
Alkaline phosphatase	74 IU/L	44-147 IU/L	
Albumin	3.1 g/dL	3.4-5.4 g/dL	
Total bilirubin	1.2 mg/dL	0.2-1.2 mg/dL	
PT	19 seconds	11-13 seconds	
INR	1.6	0.9-1.2	
PTT	33.0 seconds	25-35 seconds	
Lactic acid	1.14	0.5-2.2 mmol/L	
Total protein	11.6 gm/dL	6.0-8.5 gm/dL	
Albumin	3.1 gm/dL	3.5-5.5 gm/dL	
PTH	Low (PTH 9.9 pg/mL)	15-65 pg/mL	
PTH-RP	Low (PTH-RP 9.0 pg/mL)	<2.5 pg/mL	
Lambda light chain	7.9 mg/L	5.7-26.3 mg/L	
Kappa light chain	18.8 mg/L	3.3-19.4 mg/L	
Kappa/lambda ratio	2.38	0.26-1.65	
IgA	123 mg/dL	70-400 mg/dL	
IgM	24 mg/dL	40-230 mg/dL	
IgG	5,135 mg/dL	700-1,600 mg/dL	
IgE	10 IU/mL	0-100 IU/mL	
Beta-2 microglobulin	1.32 mg/L	0.6-2.4 mg/L	

The CT scan of the chest with IV contrast revealed widespread bony metastatic disease with numerous small lytic lesions throughout the spine and pelvis. There was a 1 cm lytic lesion with a pathologic fracture on the posterior right seventh rib, along with a sternomanubrial lesion. Furthermore, there was evidence of neoplastic infiltration and pathologic fracturing of the T7 vertebral body, with adjacent epidural soft tissue thickening. Additionally, there was posterior mediastinal and prevertebral soft tissue thickening anterior to T7 but no esophageal obstruction or stricture.

**Figure 1:**
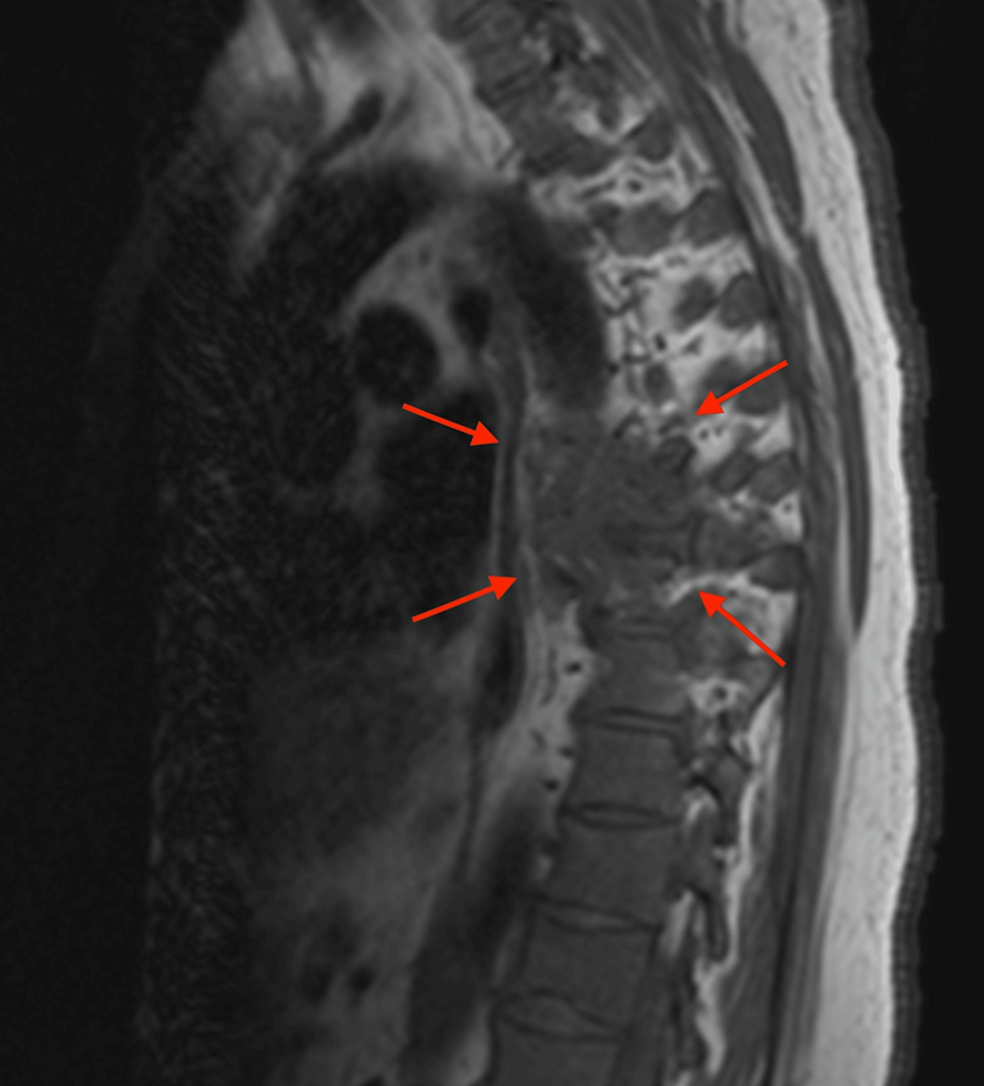
T2-weighted sagittal MRI with IV contrast showing widespread osseous metastatic neoplasm, with significant bone destruction involving the mid-thoracic spine, centered around T7

**Figure 2:**
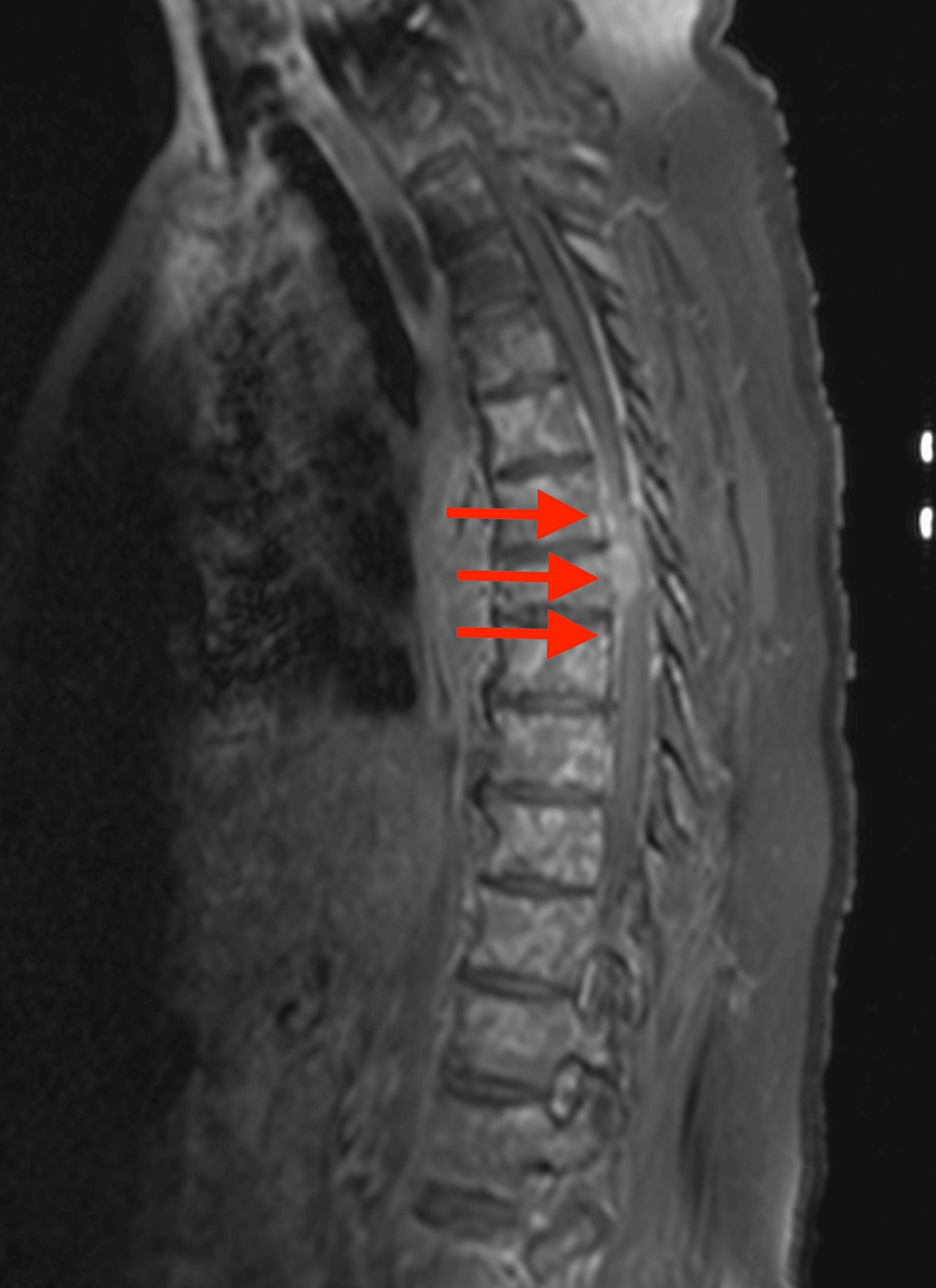
T1-weighted sagittal MRI with IV contrast showing evidence of erosion of neoplasm through the posterior endplate into the adjacent anterior epidural space, where there is associated enhancing extramedullary neoplasm with associated spinal canal narrowing

The MRI of the thoracic spine with IV contrast revealed widespread osseous metastatic neoplasm, with significant involvement in the mid-thoracic spine, centered around T7. This region shows an enhancing mass extending into the posterior mediastinum and prevertebral soft tissues, potentially contiguous with the esophagus, suggesting a primary neoplasm. Additionally, there is a pathologic fracture of the T7 vertebral body with erosion into the anterior epidural space, causing spinal canal narrowing. Dural thickening is observed cranially and caudally from this region along the thoracic spine over a 12 cm span, roughly from T4 to T9 (Figures 1-2).

**Figure 3:**
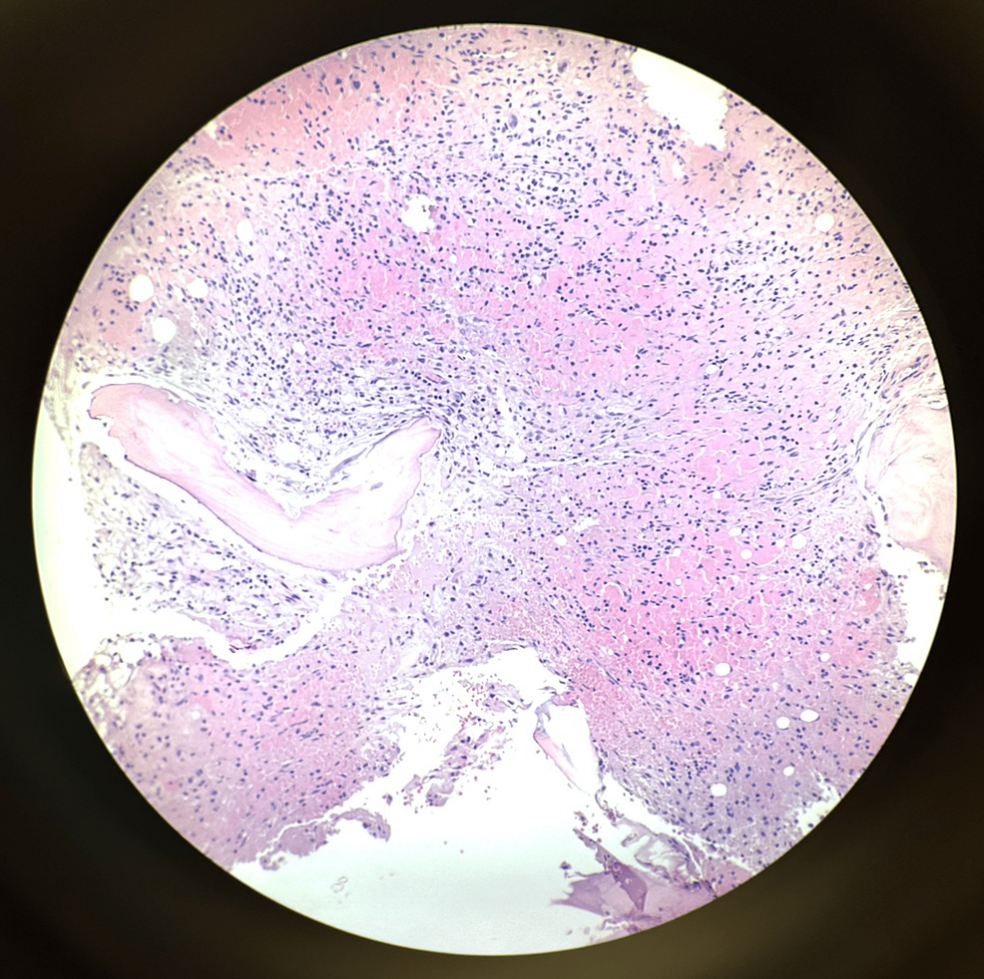
Medium power field of bone marrow smear with numerous plasma cells on H&E stain

The bone marrow biopsy revealed the presence of monoclonal kappa plasma cell infiltrates, accounting for approximately 12% of the total marrow cells, consistent with a diagnosis of plasma cell neoplasm (Figure 3).

**Figure 4:**
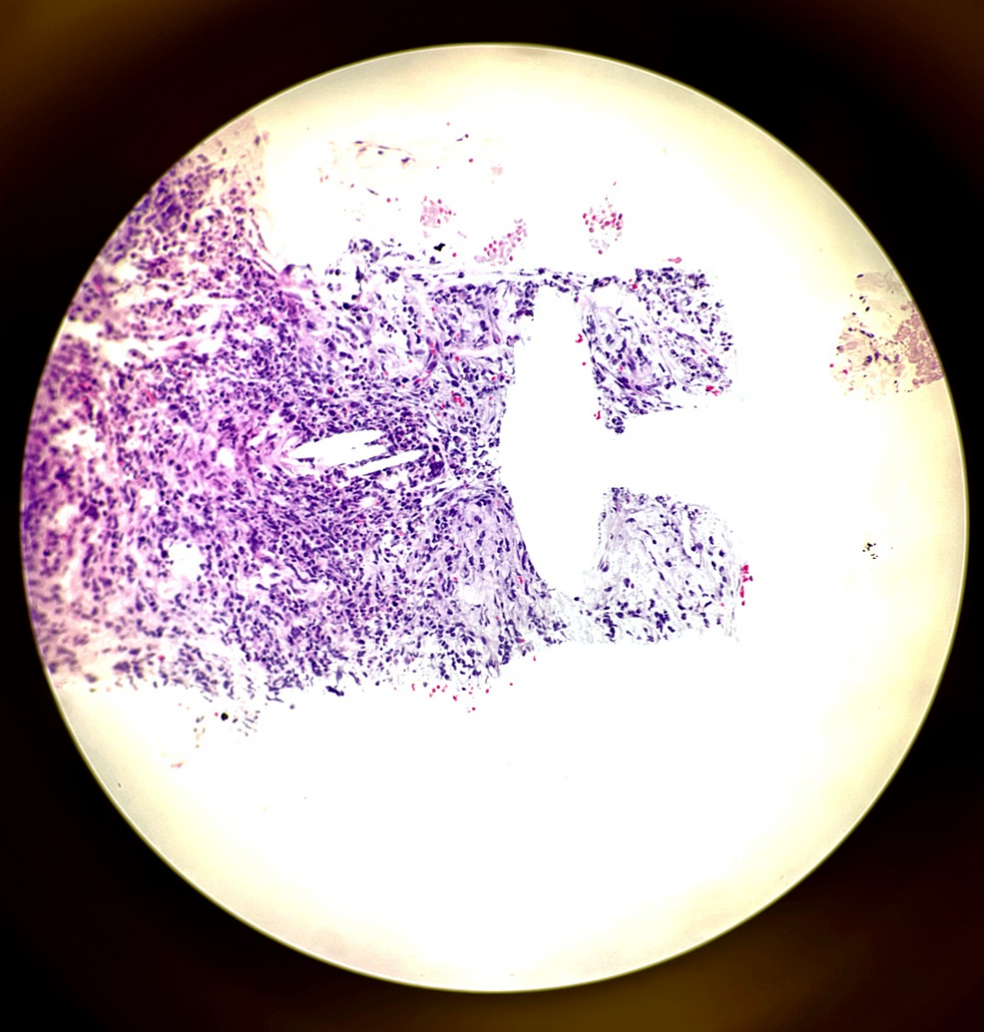
Medium power field of bone plasmacytoma on H&E stain

Notably, Congo red staining yielded negative results for amyloid deposition, and fungal stains also returned negative. Flow cytometry analysis demonstrated aberrancies in monocytes, specifically concerning CD2 and CD56 expression. Further biopsy conducted on the midthoracic mass, approximately at the T7 level, confirmed the presence of a monoclonal kappa light chain-restricted plasmacytoma (Figure 4).

Consequently, the patient received a diagnosis of stage II multiple myeloma, characterized by kappa light chain restriction and concurrent biopsy-proven bone plasmacytoma. In response to the diagnosis, the patient underwent a treatment regimen that included intravenous fluids and calcitonin administered intramuscularly every 12 hours, effectively restoring calcium levels to within the normal range. Additionally, dexamethasone was initiated at a dose of 4 mg every six hours, resulting in a significant improvement in lower extremity weakness, thus enabling the patient to regain mobility with the assistance of physical therapy.

Consultation with neurosurgery was sought, with their recommendation for surgical intervention; however, the patient declined after a thorough review of potential surgical complications. Instead, the patient opted for palliative radiotherapy, receiving a cumulative dose of 3000 cGy delivered in 10 fractions. This regimen involved external beam radiation with conformal treatment utilizing 18 mV photons with two fields, anterior and posterior, and was well-tolerated with no significant adverse effects reported.

Upon completion of the radiotherapy, the patient was discharged with appropriate arrangements for continued physical therapy. Furthermore, the patient's treatment plan included the initiation of bortezomib, lenalidomide, and dexamethasone. As a prophylactic measure against deep vein thrombosis, the patient commenced aspirin therapy with an 81 mg daily dose.

## Discussion

Plasma cell disorders encompass a diverse spectrum of conditions, ranging from precursor states to more advanced malignancies. This spectrum includes monoclonal gammopathy of undetermined significance (MGUS) and smoldering multiple myeloma (SMM), which represent earlier stages of the disease, as well as more aggressive multiple myeloma and localized plasmacytomas. In this discussion, we will explore these plasma cell disorders, their molecular intricacies, and their relevance in modern medical practice.

MGUS is characterized by the presence of a monoclonal protein in the blood, often without any symptoms or organ damage. It is considered a precursor condition that can progress to more severe conditions, including multiple myeloma. In MGUS, there is a clonal proliferation of plasma cells, but not to the extent seen in more advanced diseases. The molecular pathways underlying MGUS are still being investigated, but there are known genetic and epigenetic changes that may drive its progression. In modern management, regular monitoring is essential to detect any progression to more severe forms.

SMM represents an intermediate stage between MGUS and active multiple myeloma. In SMM, there is a higher burden of clonal plasma cells in the bone marrow compared to MGUS, but patients do not yet display the typical symptoms or organ damage seen in multiple myeloma. Molecular studies have identified specific genetic mutations and chromosomal abnormalities in SMM, such as mutations in the BRAF and KRAS genes. The management of SMM has evolved with the advent of novel therapies. Close monitoring is crucial, and treatment may be initiated earlier if there is a high risk of progression.

Multiple myeloma is a malignancy characterized by the uncontrolled proliferation of aberrant plasma cells within the bone marrow. Its pathogenesis involves a multitude of molecular pathways, notably the RAS-MAPK and PI3K-AKT signaling cascades. The treatment approach for multiple myeloma depends on the patient's eligibility for hematopoietic stem cell transplantation [4]. Eligible patients undergo induction therapy as a preparatory step for transplantation, whereas those ineligible receive several cycles of initial therapy, typically featuring a triplet regimen, followed by maintenance therapy [5]. High-dose chemotherapy, followed by autologous stem cell transplantation, remains a frequent course of action for eligible patients. Contemporary treatment modalities encompass the use of proteasome inhibitors, immunomodulatory agents, and monoclonal antibodies targeting plasma cell-specific markers. Ongoing research endeavors are concentrated on the development of precision-targeted therapies tailored to the molecular characteristics of the disease, with the overarching goal of improving clinical outcomes. Several molecular pathways and genetic abnormalities associated with multiple myeloma are detailed below (Table 2).

**Table 2:** Key molecular pathways and genetic abnormalities associated with multiple myeloma Please note that this table provides an overview of the key molecular pathways and genetic abnormalities associated with multiple myeloma. The complexity of the disease involves a combination of these factors and can vary among individual patients.

Molecular pathway	Description
RAS-MAPK pathway	Involves mutations in genes like KRAS and NRAS, activating the pathway promoting cell growth and survival.
PI3K-AKT pathway	Aberrations in the PI3K-AKT pathway, often involving PTEN mutations, increase cell survival and proliferation.
NF-κB pathway	Dysregulation of the NF-κB pathway is common, contributing to myeloma cell survival and growth.
Chromosomal abnormalities	Translocations involving the IgH locus and oncogenes like c-MYC can drive the disease.
DNA methylation	Epigenetic changes, such as DNA methylation, alter gene expression, impacting myeloma pathogenesis.
Immune evasion	Myeloma cells manipulate the immune system, often through altered expression of immune checkpoint proteins like PD-L1.
Bone marrow microenvironment	Interactions with the bone marrow microenvironment play a critical role in disease progression, impacting bone homeostasis.

Plasmacytomas are localized tumors of malignant plasma cells that can occur within bone or soft tissues. The molecular pathways in plasmacytoma are not as well defined as in multiple myeloma. Localized plasmacytomas can often be treated with radiation therapy, while systemic involvement may require systemic therapy similar to multiple myeloma.

Understanding the molecular pathways of the spectrum of plasma cell disorders is becoming increasingly relevant in modern management, guiding risk stratification and treatment decisions. Early detection and personalized therapies are key elements in improving outcomes for patients with these conditions. We will now explore the nuances of these treatment approaches.

Extramedullary plasmacytoma can be a challenging condition, particularly when it occurs in conjunction with multiple myeloma or presents as a solitary entity. The approach to treatment significantly differs between these two clinical scenarios. Extramedullary plasmacytomas may arise in patients with multiple myeloma at various points in the disease course [6].

In the case of extramedullary plasmacytoma associated with multiple myeloma, the primary focus is on managing the underlying systemic disease while addressing the local extramedullary plasmacytoma. Typically, treatment options include systemic chemotherapy regimens, such as a combination of proteasome inhibitors like bortezomib and immunomodulatory agents like lenalidomide that are often administered with corticosteroids. The goal is to achieve disease control at both the systemic and local levels. Patients may also undergo autologous stem cell transplantation as a consolidation therapy if they are deemed eligible. Radiation therapy directed at the extramedullary plasmacytoma site can be considered part of the local management. Close monitoring of disease response and regular assessments are crucial to adapting the treatment plan.

On the other hand, solitary extramedullary plasmacytoma is a localized presentation of plasma cell neoplasms. The primary treatment approach is typically radiation therapy, administered with curative intent [7]. The goal here is to achieve local control and eradicate the solitary lesion. In some cases, surgical resection may be considered if it is feasible. Unlike multiple myeloma, solitary extramedullary plasmacytoma does not require systemic chemotherapy unless there is evidence of disease progression or transformation into multiple myeloma.

It is essential to differentiate between these two scenarios as the treatment approach significantly varies, with a more aggressive approach aimed at controlling systemic disease in the presence of multiple myeloma, whereas solitary extramedullary plasmacytoma primarily involves local therapy to achieve a potential cure. Individualized treatment decisions are made based on factors such as disease extent, the patient's overall health, and the risk of progression to multiple myeloma. Close collaboration between hematologists, oncologists, and radiation oncologists is crucial to providing the most appropriate care for patients with extramedullary plasmacytoma.

Radiological imaging is fundamental in the assessment of plasma cell neoplasms. MRI, CT, and positron emission tomography scans are indispensable. These imaging modalities provide valuable insights into disease localization, extent, and characteristics [8].

In the context of solitary plasmacytoma, radiological imaging, particularly MRI, is essential for precise localization, characterization, and assessment of tumor extent within specific bones [9]. Complementary information about bone involvement and structural changes can be obtained through CT scans.

Extramedullary plasmacytomas present an added layer of complexity in the diagnosis and management of plasma cell neoplasms. Radiological techniques, including MRI and CT, are instrumental in detecting these soft tissue plasmacytomas [10]. Identifying and characterizing these extramedullary lesions is crucial for guiding treatment decisions and monitoring their response to therapy. Radiology is indispensable in this case, serving multiple critical functions as shown below (Table 3).

**Table 3:** The importance of radiology in the case

Importance of radiology	
1. Diagnosis and characterization	Radiological imaging, particularly MRI, facilitated the identification of osseous metastatic neoplasms, assessed the extent of spinal involvement, and contributed to the diagnosis of multiple myeloma with an extramedullary plasmacytoma.
2. Staging and complication assessment	Radiological findings aided in staging the disease, evaluating the extent of bone involvement, and identifying complications like pathologic fractures and spinal canal narrowing.
3. Treatment planning and monitoring	Radiology guided treatment decisions, including radiation therapy, by highlighting the location and extent of lesions. Additionally, it will continue to play a crucial role in monitoring treatment response and disease progression throughout the patient's care.

To establish a conclusive diagnosis of multiple myeloma, a bone marrow biopsy constitutes a pivotal and irreplaceable step. This procedure enables the meticulous microscopic examination of the bone marrow, facilitating the assessment of plasma cell percentages and the confirmation of the presence of aberrant plasma cells, which are distinctive hallmarks of multiple myeloma. Similarly, for the confirmation of extramedullary plasmacytoma, an indispensable requirement is a lesion biopsy to discern and characterize the anomalous plasma cells associated with this condition [11]. In cases involving spinal manifestations, specialized techniques such as fluoroscopic-guided biopsy or CT-guided lesion biopsy are harnessed, ensuring the attainment of precise and meticulously accurate tissue samples for diagnostic purposes.

## Conclusions

This case report describes the presentation, diagnosis, and management of a male patient with stage II multiple myeloma with concurrent biopsy-proven bone plasmacytoma. Understanding the molecular intricacies of these disorders, early detection, and personalized treatment strategies are pivotal in improving patient outcomes and providing the best possible care in the management of plasma cell disorders.

This case highlights the complexity of managing multiple myeloma with associated intramedullary plasmacytoma, involving a multidisciplinary approach to address both systemic and local diseases. Radiological imaging is integral to the diagnosis for accurate localization, characterization, and assessment of disease extent, guiding treatment decisions, and monitoring responses to therapy. A collaborative approach involving hematologists, oncologists, and radiologists is essential to achieve comprehensive patient care.
